# Estrogen Induction of Telomerase Activity through Regulation of the Mitogen-Activated Protein Kinase (MAPK) Dependent Pathway in Human Endometrial Cancer Cells

**DOI:** 10.1371/journal.pone.0055730

**Published:** 2013-02-07

**Authors:** Chunxiao Zhou, Tara A. Steplowski, Hallum K. Dickens, Kimberly M. Malloy, Paola A. Gehrig, John F. Boggess, Victoria L. Bae-Jump

**Affiliations:** 1 Division of Gynecological Oncology, Department of Obstetrics and Gynecology, University of North Carolina, Chapel Hill, North Carolina, United States of America; 2 Department of Otolaryngology, University of North Carolina, Chapel Hill, North Carolina, United States of America; II Università di Napoli, Italy

## Abstract

Given that prolonged exposure to estrogen and increased telomerase activity are associated with endometrial carcinogenesis, our objective was to evaluate the interaction between the MAPK pathway and estrogen induction of telomerase activity in endometrial cancer cells. Estradiol (E2) induced telomerase activity and hTERT mRNA expression in the estrogen receptor (ER)-α positive, Ishikawa endometrial cancer cell line. UO126, a highly selective inhibitor of MEK1/MEK2, inhibited telomerase activity and hTERT mRNA expression induced by E2. Similar results were also found after transfection with ERK 1/2-specific siRNA. Treatment with E2 resulted in rapid phosphorylation of p44/42 MAPK and increased MAPK activity which was abolished by UO126. The hTERT promoter contains two estrogen response elements (EREs), and luciferase assays demonstrate that these EREs are activated by E2. Exposure to UO126 or ERK 1/2-specific siRNA in combination with E2 counteracted the stimulatory effect of E2 on luciferase activity from these EREs. These findings suggest that E2-induction of telomerase activity is mediated via the MAPK pathway in human endometrial cancer cells.

## Introduction

Endometrial cancer is the most most common malignancy in women in the United States [1]. Endogenous and exogenous estrogen exposure are major risk factors for the development of type I endometrial cancers; however, the molecular link between estrogen and endometrial carcinogenesis remains poorly understood. Our previous work demonstrated that estrogen regulation of telomerase may potentially play a role in the malignant transformation of the endometrium [2].

Telomeres are specialized structures of the distal end of chromosomes and function in chromosome protection, positioning, and replication. With aging, human telomeres inevitably undergo progressive shortening in normal somatic cells through the replication-dependent sequence loss at terminal ends of the DNA. The progressive shortening of telomeres eventually results in chromosomal instability, leading to cellular senescence. Telomerase is a ribonucleoprotein reverse transcriptase that synthesizes telomeric DNA into chromosomal ends. This enzyme recognizes the G-rich strand of an existing telomere repeat sequence and synthesizes a new copy of the repeat sequence in the absence of a complementary DNA strand, with a segment of its internal RNA component serving as a template [3], [4]. Thus, telomerase is comprised of an RNA template (human telomerase RNA, hTR) and the catalytic protein hTERT (human telomerase reverse transcriptase, hTERT) which has reverse transcriptase activity [5]–[7]. The expression of hTERT is observed at high levels in telomerase-positive cancer cells but not in telomerase-negative cells, and is considered the rate-limiting determinant of telomerase activity [7], [8].

More than 85% of human endometrial carcinomas express telomerase activity [9]–[12], and the level of telomerase activity has been correlated with advanced stage disease and with pelvic lymph node metastasis [12]. The human endometrium is a uniquely dynamic tissue, consisting of epithelial glands and connective tissue that undergoes complex patterns of proliferation, secretion, and breakdown throughout the reproductive years. During the menstrual cycle, endometrial epithelial cells are regulated by the sex hormones estrogen and progesterone, and endometrial carcionogenesis is thought to be associated with prolonged exposure to estrogen, unopposed by progesterone. In the normal endometrium, expression of telomerase is correlated with cellular proliferation, is typically localized in epithelial glandular cells, and is regulated in a hormonally-driven, menstrual phase-dependent manner [13], [14]. Increased telomerase activity is observed in the proliferative phase when estrogen levels are maximal followed by near absent levels in the secretory phase when progesterone levels are high [13]. Such evidence suggests a relationship between sex steroid levels, the modulation of telomerase activity and the development of endometrial cancer.

The promoter region of hTERT has been cloned and characterized and contains two putative estrogen response elements (EREs), implying a direct linkage between estrogen and telomerase regulation [2], [15]–[18]. We have previously found that telomerase activity and hTERT mRNA were increased in response to estrogen in an estrogen receptor-α (ERα) dependent fashion in endometrial cancer cell lines [2]. Furthermore, we demonstrated binding of complexed estrogen with ERα to the EREs found within the hTERT promoter, indicative of a possible underlying mechanism between telomerase induction and the malignant transformation of hormone-dependent endometrial cells [2].

Mitogen-activated protein kinases (MAPK) are an important family of protein kinases involved in transmitting signals from the cell membrane to the nucleus. It is well known that the p44/42 MAPK signaling pathway is activated by mitogenic stimuli from growth factors and sex steroid hormones such as estrogen, progesterone, and epidermal growth factor (EGF) in human breast, ovarian and endometrial cancer cells, among others [19]–[21]. In order to gain insight into the molecular mechanisms that control the regulation of telomerase activity by estrogen, we examined the relationship between the MAPK pathway and estrogen-induced telomerase activity in human endometrial cancer cells.

## Materials and Methods

### Cell Culture and Reagents

The regulation of telomerase expression was investigated in ER-positive (Ishikawa) and ER-negative (HEC-1B) human endometrial cancer cell lines [22], [23]. These cell lines were provided as gift by Dr. Bruce Lessey (Center for Women's Medicine, Greenville, SC). As previously described, estrogen-induced ERE chloramphenicol acetyltransferase (CAT) activity was determined in each of these cell lines to confirm functional ER status [24]. Ishikawa and HEC-1B cells were grown in MEM supplemented with 5% fetal bovine serum (FBS), 100 units/ml penicillin and 100 µg/ml streptomycin in the presence 5% CO_2_ at 37°C. The endometrial cancer cell lines were cultured in phenol-red free medium with 0.5% charcoal-dextran-treated FBS for 1 day before treatment with estrogen or U0126.

### Chemicals and plasmid

All hTERT reporter promoter luciferase plasmids were provided by Dr. I. Horikawa (National Institute of Health, Bethesda, MD). 17-β estradiol (E2) was purchased from Sigma (St. Louis, MO). UO126 was purchased from Calbiochem (La Jolla, CA). The anti-phosphorylated p42/44 and anti-nonphosphorylated p42/44 antibodies were from Cell Signaling Technology (Beverly, MA). The anti-β-actin antibody was from Santa Cruz Biotechnology (Santa Cruz, CA). The enhanced chemiluminescence Western blotting detection reagents were from Amersham (Arlington Heights, IL). All other chemicals were from Sigma (St. Louis, MO).

### E2 and U0126 Treatment

Cells were seeded at 4×10^5^ cells per T25 culture flask or 1.5×10^5^ cells per well of a 12-well plate, containing either 5 ml or 1.2 ml regular medium, respectively. The media was then changed to phenol-red free medium with 0.5% stripped FBS for incubation at 37°C overnight. Immediately prior to treatment, the medium in the culture plates was aspirated, triply washed with phosphate-buffered saline (PBS) and replaced with fresh medium. E2 dissolved in ethyl alcohol or U0126 dissolved in DMSO was subsequently added to each well. Concurrently, the same amount of ethyl alcohol or DMSO was added to the control wells.

### Telomerase Activity Assay

Telomerase activity was measured using a PCR-based telomeric repeat amplification protocol (TRAP) (TRAP-eze telomerase detection kit, oncor, Gaithersburg, MA). Briefly, cell pellets were lysed, homogenized with 105 uL of ice-cold 1× CHAPS, put on ice for 30 min, and centrifuged at 13000 g for 21 min at 4°C. The resulting supernatent was then transferred into a fresh tube and stored at −80°C. A sample from the extract was then taken and the protein concentration was determined using the BCA kit (Bio-Rad, Hercules, CA). Between 0.25 and 05 ug of protein, placed in a 50-µL reaction mixture, was used for the TRAP assay. After 30 min of incubation at 30°C, 27 cycles of PCR amplification were performed (30 min at 94°C followed by 30 min at 59°C). The PCR products were then analyzed by electrophoresis on 10% polyacrylamide non-denaturing gels. Gels were analyzed and quantified using the PhosphorImaging system with Imagequant software (Molecular Dynamics inc., Sunnyvale, CA). Each experiment was performed twice.

### Real-time RT-PCR for hTERT

Total RNA was isolated using the RNAqueous kit (Ambion, Austin, TX) and further purified using the DNA-free kit (Ambion, Austin, TX). The reverse transcription and PCR reactions were performed using the TaqMan Gold one-step RT-PCR kit in the ABI Prism 7700 Sequence Detection System (Applied Biosystems, Foster City, CA). Reverse transcription was carried out at 48°C for 30 min. The PCR conditions consisted of a 10-min step at 95°C and 40 cycles between 95°C for 15 s and 65°C for 1 min. A housekeeping control gene, acidic ribosomal phosphoprotein P0 (RPLP0, also known as 36B4), was used as an internal control to correct for differences in the amount of RNA in each sample [25]. Primers and fluorogenic probes for hTERT and RPLP0 have been described previously [25]. The standard curve for hTERT was generated by using dilutions of a known amount of cRNA synthesized by *in vitro* transcription of a cloned fragment. The normalized level of hTERT in each sample was estimated by a ratio of the hTERT level to the RPLP0 level, as described previously [25]. Experiments were performed in duplicate and repeated twice for consistency.

### Western Blot Analysis

Ishikawa cells were plated at a density of 3×10^5^ cells/well in six-well plates. After 24 hours, the medium was aspirated and replaced with serum-free medium. After 24 hours, the cells were treated with E2, UO126 or both in combination for a minimum of 15 min and a maximum of 24 hr. The plates were scraped with RIPA buffer and cell lysates were prepared in 2× SDS buffer. The cell lysates were separated by 12% SDS-PAGE gel and transferred onto a nitrocellulose membrane. The membrane was blocked for 1 hr in TBS-T +5% nonfat dry milk, and then incubated with phosphophorylated-p44/42 MAPK monoclonal antibody (1∶2000) or pan-p44/42 MAPK polyclonal antibody (1∶1000) overnight at 4°C (Cell Signaling, Beverly, MA). The membrane was then washed in TBS-T and incubated with a secondary peroxidase-conjugated antibody for 1 hr. Antibody binding was detected using an enhanced chemiluminescence detection system (ECL). Band net intensities were quantified using a Millipore Digital Bioimaging System (Bedford, MA). Each experiment was performed twice.

### *In Vitro* Kinase Assay

Activity of p44/42 kinase activity was measured *in vitro* using the p44/42 MAP kinase assay kit (Cell Signaling, Danvers, MA). Briefly, Ishikawa cells were plated at a density of 3×10^5^ cells/well in six-well plates. Following E2 and UO126 treatment, plates were washed 4 times with ice-cold PBS, and 0.5 mL ice-cold lysis buffer +1 mM PMSF was added to each well. Cells were scraped, sonicated, and centrifuged at 10,000 g for 10 min at 4°C. The resulting supernatent was incubated with 15 uL of resuspended immobilized phospho-p44/42 MAP kinase (Thr202/Tyr204) monoclonal antibody for 12 hr at 4°C. The immune complex was washed with lysis buffer 5 times and once with kinase buffer without substrate. Immunoprecipitated MAPK was then incubated for 30 min at 30°C in kinase buffer with 2 ug Elk1 fusion protein and 200 uM ATP. The kinase reaction was stopped by adding 25 ul of 3× SDS buffer. ElK1 was separated by SDS-PAGE gel, and was then incubated with anti-phospho-Elk1 (Ser 308) antibody at 4°C overnight. Elk1 was detected with ECL as described for western blot analysis. Each experiment was performed twice.

### Luciferase assay

Transient transfection of luciferase reporter plasmids was performed using the TransFast Transfection Reagent (Promega, Madison, WI). Briefly, 8×10^4^ of Ishikawa cells were seeded in 24-well plates overnight and transfected with promoter luciferase plasmids (0.5 µg/well). The pRL-SV40 (2 ng/well) containing Renilla reniformis luciferase was co-transfected in each transfection as an internal control to normalize the transcriptional activity of the hTERT promoter plasmids. The luciferase assay was then performed using the Dual Luciferase Reporter Assay System (Promega, Madison, WI) according to protocols provided by the manufacturer. All experiments were performed in triplicate for each plasmid, and each experiment was performed twice.

### ERK 1/2 siRNA assay

ERK 1/2 small interfering RNA (siRNA) was purchased from Cell Signaling Technology (Danvers, MA). According to the manufacturer's instructions, Ishikawa cells were plated in 6-well plates or 12-well plates at the recommended cell concentration. After 24 hours, transfections were performed at approximately 60% confluency using transfection reagent (Santa Cruz Biotechnology, Santa Cruz, CA). For each transfection reaction, 100 nM ERK 1/2 siRNA or control siRNA was used for preparation of siRNA-transfection complexes at room temperature for 15 min. Transfections were performed in 0.5 (12-well plate) or 1.5 mL (6-well plate) serum-free medium for 8 hr. After incubation, transfection complexes were removed and replaced with their corresponding media. In each experiment, untreated controls receiving transfection reagent were included. Transfection efficiency (80%–90%) was determined by fluorescence microscope in fluorescein-labeled nonspecific siRNA transfected cells and by Western blotting analysis. Cells were utilized for Western blotting analysis, TRAP assay, lucifersae activity and real time PCR at 36–48 hr after transfection.

## Results

### The effect of E2 on hTERT mRNA and telomerase activity in Ishikawa cells

**Figure 1:**
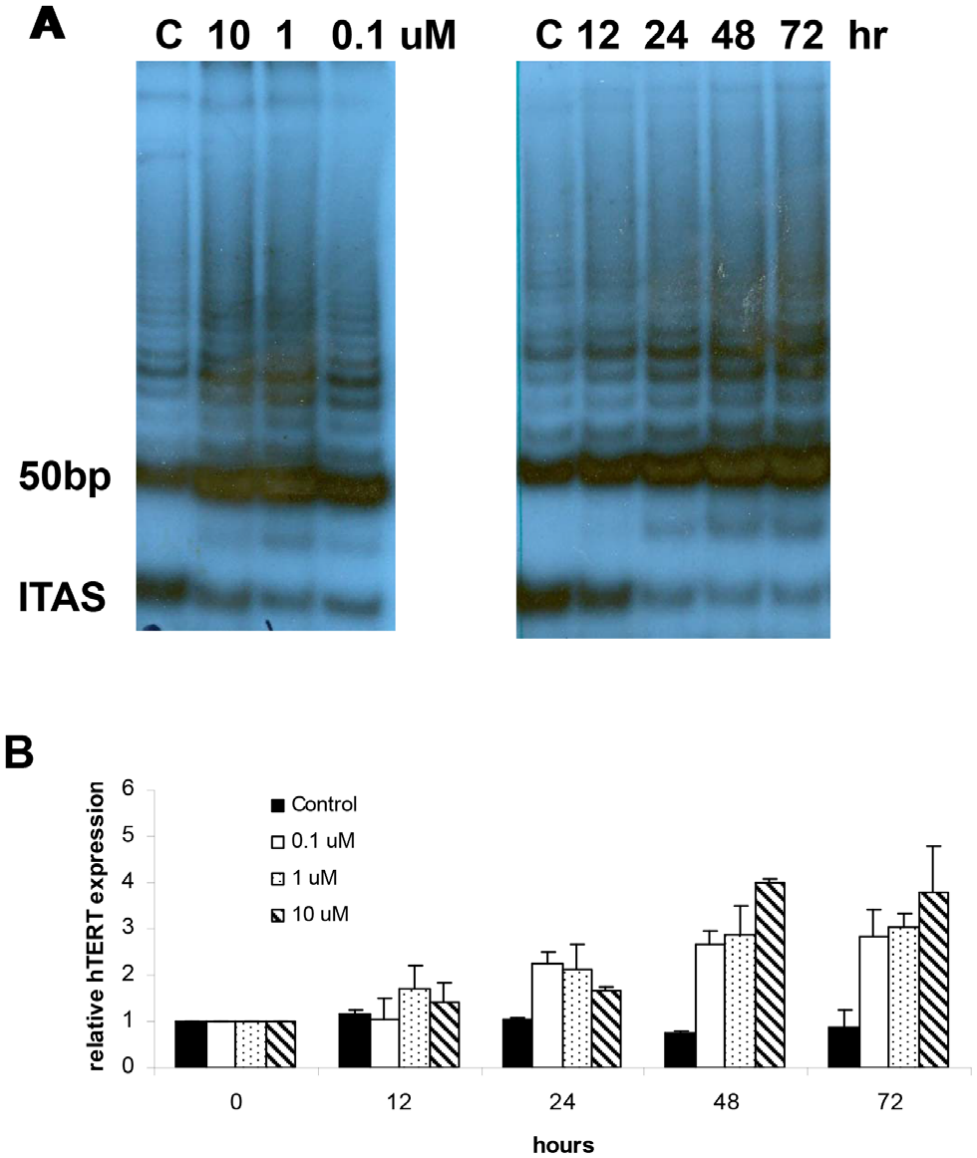
The effect of E2 on telomerase activity and hTERT expression in the ER-positive Ishikawa cell line. Cells were treated with different concentrations of E2 (0.1–10 µM) for 48 hr or treated with 1 uM E2 in a time-course fashion (A). Telomerase activity was determined by the TRAP assay. hTERT RNA expression was assessed by real-time RT-PCR (B). The data are presented as means ± SD of duplicated samples from at least two independent experiments. (ITAS = internal telomerase assay standard, C = control).

In the Ishikawa cell line, E2 was found to increase telomerase activity in a dose-dependent manner as assessed by TRAP assay (Figure 1A). The increased activity was dependent on both E2 concentration and length of time of exposure. When 0.1–10 µM of E2 was added, telomerase activity was up-regulated at 12 hr, which persisted until at least 72 hr following treatment. Treating cells with 1–10 µM of E2 for more than 48 hr induced maximal telomerase activity. No effect of E2 on telomerase activity was detected in the ERα negative endometrial cancer cell line (HEC-1B), under the same treatment conditions (data not shown).

To understand the underlying mechanism of induction of telomerase activity, we quantified by real-time RT-PCR assay the level of hTERT mRNA expression under the same conditions. The hTERT gene encodes the catalytic subunit of telomerase and is usually the rate-limiting determinant of telomerase enzymatic activity. E2 increased hTERT mRNA expression in a dose-dependent manner (1–10 µM). This up-regulation of activity was observed within 6 hr after E2 treatment, and maximal induction of hTERT mRNA expression was observed at 48 hr. E2-induced hTERT mRNA expression remained elevated for greater than 72 hr (Figure 1B). These findings suggest that the regulation of telomerase activity by E2 may occur at the transcriptional level in endometrial cancer cells.

### The effect of UO126 on telomerase activity and hTERT mRNA expression in Ishikawa cells

**Figure 2:**
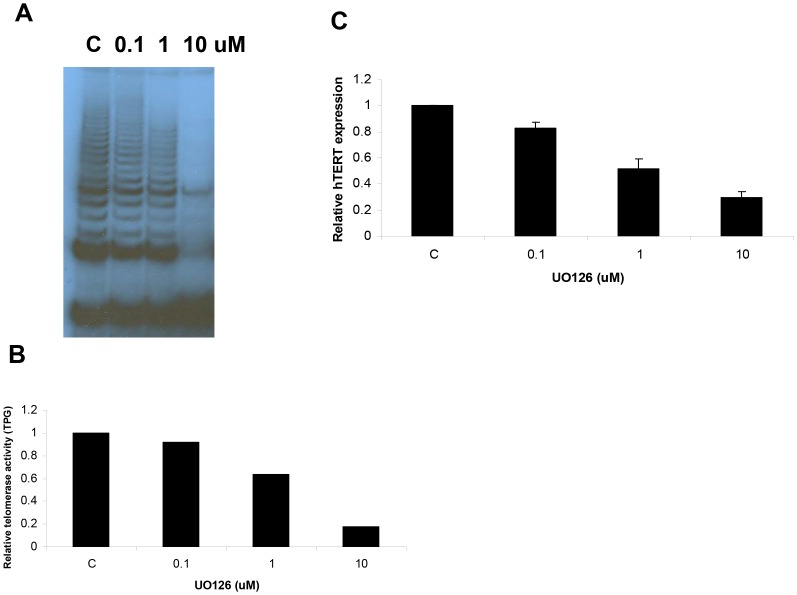
The effect of UO126 on telomerase activity and hTERT mRNA expression. The Ishikawa cells were treated with UO126 at varying concentrations (0.1–10 µM) for 24 hr. Telomerase activity was assessed by TRAP assay (A and B) hTERT expression was assessed by real time RT-PCR (C). (C = control).

To address the role of the MAPK pathway in E2-induced telomerase activity in Ishikawa cells, we initially assessed the ability of UO126, a specific MEK1 and MEK2 inhibitor, to directly inhibit telomerase activity. In the Ishikawa cells, UO126 inhibited telomerase activity in a dose-dependent manner (0.1–10 µM) as demonstrated by TRAP assay (Figure 2A and 2B). In parallel, UO126 decreased hTERT mRNA expression in a dose-dependent manner, as quantified by real-time RT-PCR (Figure 2C). These results demonstrate that UO126 is sufficient to inhibit telomerase activity and hTERT mRNA transcription in endometrial cancer cells.

### The effect of UO126 on E2-induced telomerase activity and hTERT mRNA expression in Ishikawa cells

**Figure 3:**
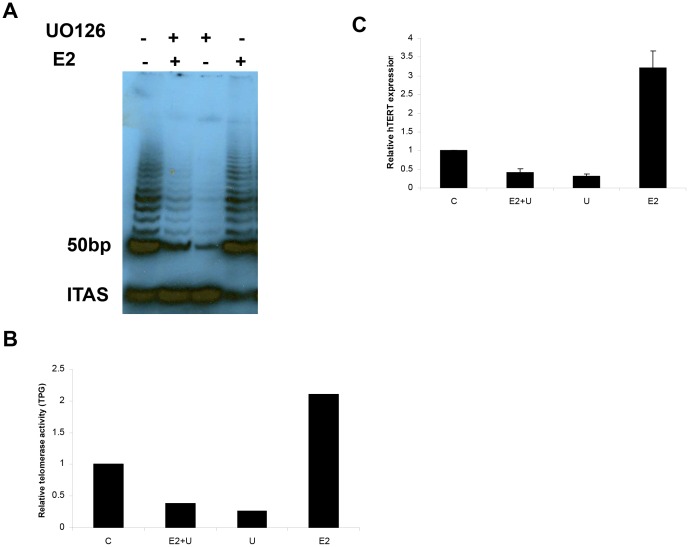
The effects of UO126 in combination with E2 on telomerase and hTERT mRNA expression in Ishikawa cells. Cells were treated with 10 uM UO126 (U), 1 uM E2 or both in combination for 24 hr. (A) Telomerase activity was assessed by TRAP assay. (B) Telomerase activity represented in graphical from using TPG (total product generated) which corresponds to relative telomerase activity. TPG is calculated from the ratio of TRAP product band to the internal telomerase assay standard band. (C) hTERT mRNA expression was determined by real time RT-PCR. (C = control).

To investigate the impact of UO126 on E2-induced telomerase activity and hTERT mRNA expression, cells were treated with 10 µM of UO126, 1 µM of E2, or both in combination (10 µM UO126+1 µM E2) for 24 hr, and telomerase activity and hTERT expression was subsequently determined. As shown in Figure 3, E2 induced increased telomerase activity and hTERT mRNA expression to approximately 2–3 fold that of controls, and these stimulatory effects were nearly abolished in the presence of UO126 (10 µM). These data suggest that the function of the MEK inhibitor, UO126, occurs not only at the level of the telomerase enzyme itself, but also at the transcriptional level of the hTERT gene.

### The effect of UO126 on E2-induced activation of p44/42

**Figure 4:**
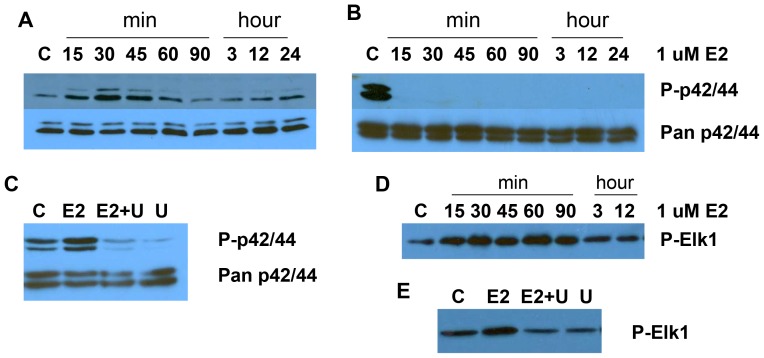
The effects of E2 and UO126 on phosphorylation of p42/44 and ERK1 kinase activity in Ishikawa cells. Cells were treated with 1 uM E2, 10 uM UO126 (U) or both in combination for a minimum of 15 min and a maximum of 24 hr. Phosphorylation of p42/44 was determined by Western blotting analysis. MAPK activity was assessed by immunoprecipitation assay for Elk1. E2 induced phosphorylation of p42/44 (A) and MAPK activity in a time-dependent fashion (D). UO126 inhibited phosphorylation of p42/44 and MAPK activity induced by E2 (B, C and E). (C = control).

In order to assess the interaction between E2 and the MAPK pathway, we used a phosphorylated-specific p44/42 MAPK antibody to identify E2-induced tyrosine phosphorylation of these kinases in the Ishikawa cell line. Under serum-starved conditions, we observed a low basal level of tyrosine-phosphorylated forms of p44/42. Following treatment with 1 µM E2, phosphorylation of p44/42 was rapidly induced and reached maximal induction 30 min after treatment. The level of phosphorylation of p44/42 remained elevated for approximately 60 min (Figure 4A). The total p44/42 MAPK protein level remained constant throughout these experiments, as determined by using a non-phosphorylated antibody to p44/42 for the same cell membranes. Incubation with 10 µM UO126 completely blocked E2-induced phosphorylation of p44/42 (Figure 4B and 4C).

To confirm that the increased phosphorylation of p44/42 represented the enzymatic activity of the protein, we measured p44/42 kinase activity via an immune complex kinase assay in which Elk1 served as the substrate. The Elk1 transcription factor is a well-known downstream target of p44/42 and considered a marker of MAPK activity. We observed a measurable increase in the activity of p44/42 after E2 stimulation, with a maximal peak at 30 min. The p44/42 kinase activity was proportional to the degree of phosphorylation of p44/42. UO126 also decreased the p44/42 kinase activity induced by E2 (Figure 4D and 4E). These data confirm a relationship between E2 signaling and the MAPK pathway in the Ishikawa cell line.

### The effect of E2 and UO126 on hTERT promoter activity

**Figure 5:**
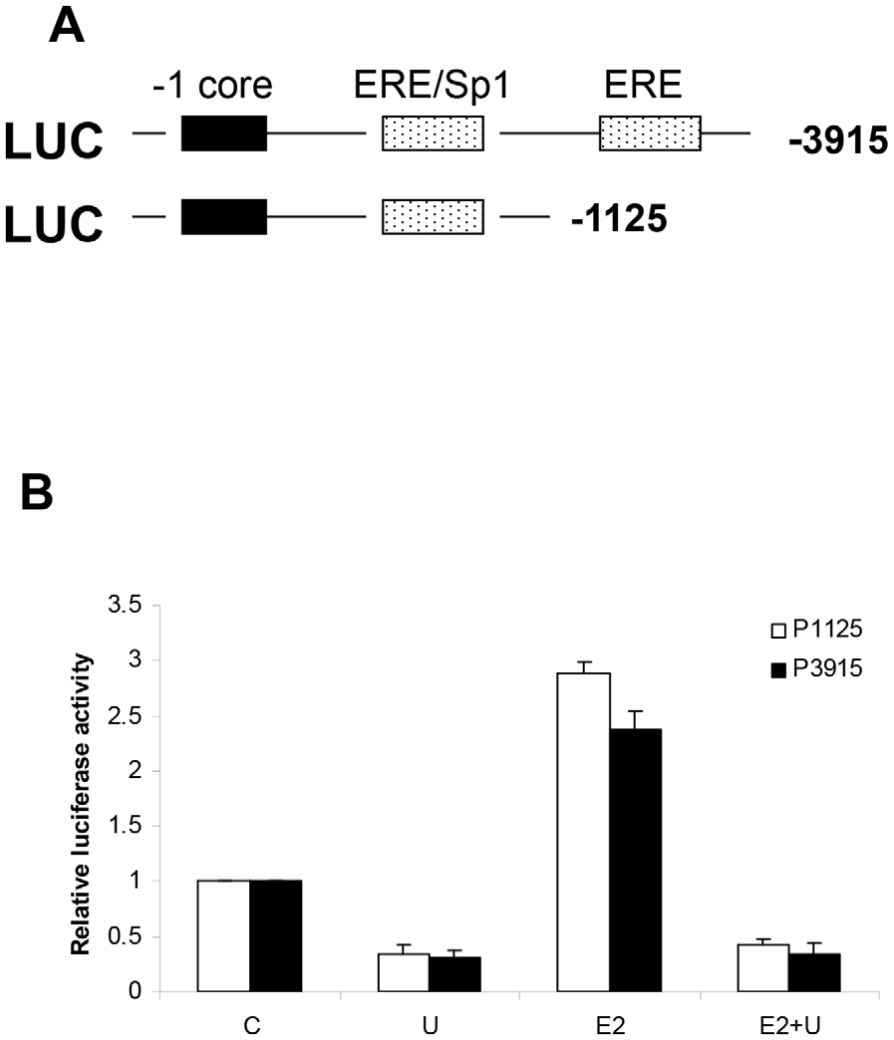
The effect of estrogen and UO126 on hTERT promoter activity. Schematic diagram of hTERT promoter luciferase plasmids showing two ERE binding sites and core promoter (A). Ishikawa cells were transfected with hTERT promoter luciferase plasmids and luciferase activity was assayed after exposure to 1 uM E2 or 10 uM UO126 (U) for 36 hr. The data are presented as means ± SD of duplicated samples from at least two independent experiments. (C = control).

Based on our previous work in endometrial cancer cell lines [2], the transcriptional activity of the hTERT promoter may be mediated by ERα binding to EREs located on this promoter. The regulatory promoter sequence of the hTERT gene contains two putative EREs in the 5′ flanking sequence: the distal one at −2777/−2755 and the proximal one at −979/−956 that overlaps with an Sp1 binding site [16], [17]. To determine if UO126 is involved in regulating hTERT promoter activity induced by E2, we performed a series of transient transfections with luciferase reporter plasmids containing the varying lengths of the 5′ promoter region of the hTERT gene. In brief, the Ishikawa cells were transfected with two ERE-responsive reporters, one of which contained both ERE sites, and the other which contained only the proximal ERE/Sp1 site (Figure 5A). As shown in Figure 5B, E2 increased luciferase activity from both reporters to approximately 2.5 to 3 fold that of the control. UO126 effectively blocked luciferase activity from these reporters in the presence of E2 (Figure 5B). These results suggest that the MAPK pathway is involved in E2/ERα-activation of the EREs in the hTERT promoter; and thus, this pathway may be critical in mediating hTERT transcriptional activity induced by E2.

### The effects of ERK1/2-specific siRNA in combination with E2 on telomerase activity, hTERT expression and hTERT promoter activity in Ishikawa cells

**Figure 6:**
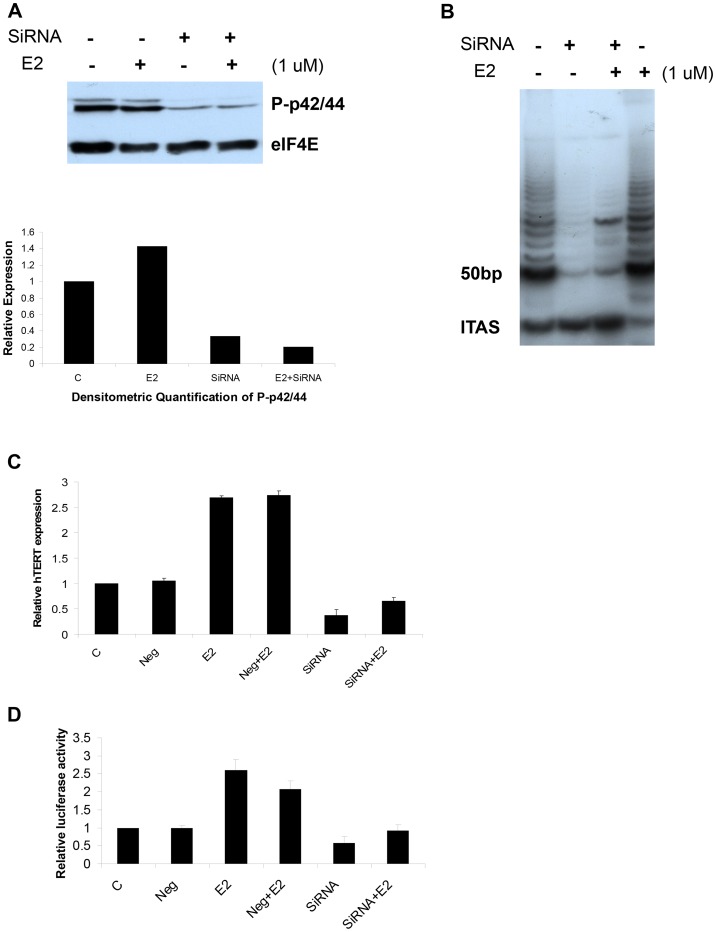
The effects of ERK1/2-specific siRNA in combination with estrogen on telomerase, hTERT expression and hTERT promoter activity in Ishikawa cells. The cells were either transfected with ERK1/2 siRNA or negative control (Neg) for 8 hr and then treated with 1 uM estrogen for 36–48 hr. The effect of transfection with ERK 1/2-specfic siRNA on phosphorylation for p42/p44 induced by estrogen was assessed by Western blotting at 48 hr (Figure 6A). Telomerase activity and hTERT expression were assayed by TRAP assay and real time RT-PCR at 48 hr (Figure 6B and 6C). Lucifersae activity was assayed after cells were transfected with ERK1/2 siRNA for 24 hr, followed by transfection with the hTERT promoter luciferase plasmid (P3915) and then treatment with 1 uM estrogen for 36 hr (Figure 6D). (ITAS = internal telomerase assay standard, C = control).

In order to verify the involvement of the MAPK pathway in the E2 induction of telomerase activity, we examined the consequences of transfection with ERK 1/2-specific siRNA in combination with E2 on telomerase activity, hTERT expression and hTERT promoter activity in the Ishikawa cell line. ERK1/2-specific siRNA reduced phosphorylation of p42/44 induced by estrogen at 48 hr, as determined by Western blotting analysis (Figure 6A). By densitometric quantification normalized to control protein eIF4E, E2 increased phosphorylation of p42/44 by 29%, and E2 in the presence of ERK1/2 specific siRNA decreased phosphorylation of p42/44 by 79%. Telomerase activity and hTERT expression were assayed by TRAP assay and real time RT-PCR at 48 hr (Figure 6B and 6C). Similar to treatment with U0126, E2 induced increased telomerase activity and hTERT mRNA expression to approximately 2–3 fold that of controls, and these stimulatory effects were nearly abolished in the presence of ERK 1/2-specific siRNA. Lucifersae activity was assayed after cells were transfected with ERK1/2 siRNA for 24 hr, followed by transfection with the hTERT promoter luciferase plasmid (P3915) and then treatment with 1 uM estrogen for 36 hr (Figure 6D). As found for UO126, transfection with ERK 1/2-specific siRNA effectively blocked luciferase activity from the P3915 promoter in the presence of E2. These results provide further evidence of the inter-relationship between the MAPK pathway and E2-mediated induction of hTERT transcriptional activity.

## Discussion

We have previously shown that E2 induces telomerase activity and hTERT mRNA expression in ER-positive endometrial cancer cell lines, potentially through binding of complexed estrogen with ERα to EREs found within the hTERT promoter. In the present study, we wanted to elicit the underlying molecular mechanism involved in regulating telomerase activity and hTERT mRNA expression induced by E2 in the ERα-positive Ishikawa cell line. Given the well-established relationship between ERα and the MAPK pathway, it seemed logical that this pathway may be involved in the induction of telomerase by E2.

Treatment with UO126, a highly selective inhibitor of both MEK1 and MEK2, inhibited telomerase activity and hTERT mRNA expression in the Ishikawa cells. In addition, U0126 completely blocked E2-stimulated telomerase activity and hTERT mRNA expression. Similar results were also found after transfection with ERK 1/2 –specific siRNA. Treatment with E2 resulted in rapid phosphorylation of p44/42 MAPK and increased MAPK functional activity, and this effect could be abolished by the addition of UO126. Luciferase assays demonstrate that exposure to UO126 or ERK 1/2-specific siRNA counteracted the stimulatory effect of E2 treatment on EREs located in the hTERT promoter. Thus, we provide evidence that E2 induces telomerase activity and hTERT mRNA expression in ERα-positive endometrial cancer cells that is dependent on signaling through the MAPK pathway. To our knowledge, only one other study has implicated a cooperative role for MAPK in the regulation of hTERT with the ER, and this was specifically for ERβ in human pancreatic cell lines [26].

A variety of studies have shown that the response of target genes to estrogen depends on several important factors including the nature of the estrogen receptor, the ligand and cell context, target gene promoter and specific transcriptional factors, as well as agents affecting protein kinase activation and protein phosphorylation [2], [16], [27]–[29]. The human hTERT promoter contains an imperfect estrogen response element (ERE) and an ERE/Sp1 half site. We and others have found that estrogen-induced hTERT gene expression and telomerase activity may result from the direct binding of ERα to two ERE sites located in the promoter region of the hTERT gene [2], [15]–[17]. In addition to EREs, this region possesses consensus sequences for the binding of transcription factors such as Sp1, Ap2, Ap4, c-Myc, NF-1 and E-box [8], [29]–[31]. It has been demonstrated that E2 activation of the core hTERT promoter can be completely eliminated when the c-Myc sites are abrogated by mutations [16]. E2 has also been found to significantly activate the c-Myc promoter in luciferase assays using c-Myc promoter-reporter plasmids [16]. Based on this culminated evidence, it can be postulated that estrogen may mediate hTERT transcriptional activity through ERα directly binding EREs in the hTERT promoter or alternatively, by E2-activation of transcription factors that interact with this promoter, or most likely via a combination of both mechanisms.

The MAPK signaling cascade regulates a variety of cellular activities, including cell growth, differentiation, survival and cell death. This pathway is thought to be essential in the regulation of ERα transcription, including a novel mechanism by which ERα and ERK2 co-localize at chromatin binding sites and collaborate in the regulation of hormone stimulation of proliferation [32], [33]. Estrogen receptor signaling and activation of the MAPK pathway have also been implicated in the development and progression of hormonally-driven cancers, such as breast and endometrial cancer. Progesterone has been found to inhibit the estrogen-induced activation of telomerase activity in breast and endometrial cancer cell lines [34], and the MAPK pathway was demonstrated to be partially responsible for this antagonistic effect. Tamoxifen, a common adjuvant therapy for breast cancer, has estrogen-like properties in uterine tissue and is associated with an increased risk of endometrial cancer. Treatment of endometrial cancer cells with tamoxifen has been shown to lead to the induction of telomerase activity which could be effectively blocked by a MEK inhibitor. Sp1 binding sites and ETS family member motifs have been found in the hTERT promoter 5′-flanking sequence [30], [35]–[37], and these represent potential transcription factors that are targeted by the MAPK pathway and may be induced through estrogen and progesterone signaling. MAPK cascade-mediated histone phosphorylation has also been implicated in transcriptional activation of the hTERT gene in normal and malignant cells [38]. Our work provides further evidence of the link between the MAPK pathway and hormonal regulation of hTERT gene transcription.

Although UO126 and ERK 1/2 siRNA were both very effective in blocking E2-induced telomerase activity and hTERT mRNA, some residual activity did remain. This suggests that cell signaling pathways other than the MAPK pathway may play a role in E2-regulation of telomerase, such as the PI3K/Akt/mTOR pathway. In ovarian cancer cell lines, E2 has been found to stimulate telomerase activity via two mechanisms – transcriptional regulation of hTERT via an ERE-dependent mechanism and the PI3K/Akt/mTOR cascade as well as post-translational regulation through Akt-dependent phosphorylation of hTERT [15]. Thus, in parallel to E2's complex effects on proliferation of the endometrium, the interplay of E2 on telomerase activity may also involve multiple signaling cascades.

**Figure 7:**
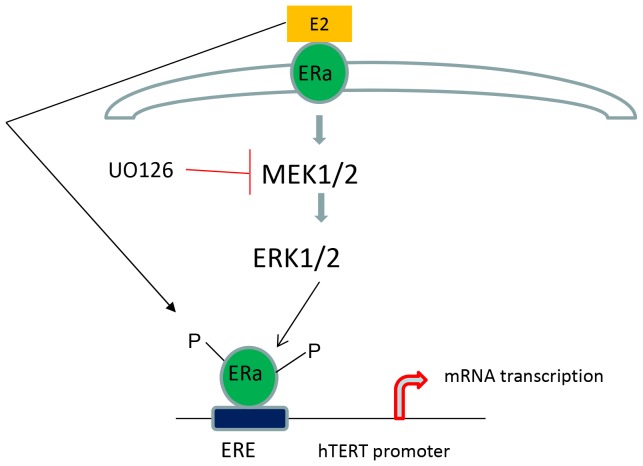
Proposed model of the interaction between E2 and the MAPK pathway for the regulation of telomerase activity. In essence, exposure to E2 leads to phosphorylation of p42/44 which is necessary for hTERT mRNA expression. However, MAPK activation is also critical for transactivation of the hTERT promoter by E2.

In summary, our data suggest that estrogen induces telomerase activity at the hTERT transcriptional level in endometrial carcinoma cells, and this effect is mediated via the interaction between estrogen and the MAPK pathway. To our knowledge, this is the first study to link estrogen regulation of telomerase activity with signaling through the MAPK pathway. We speculate that both the estrogen-ERα-system-mediated and the MAPK-pathway–mediated regulation of telomerase activity may be related through a positive feedback mechanism, as depicted in the model found in Figure 7. Our findings support cross talk between E2, the MAPK pathway and regulation of telomerase activity. As shown in this model, exposure to E2 leads to phosphorylation of p42/44 which is necessary for hTERT expression. However, MAPK activation is also critical for transactivation of the hTERT promoter by E2. Further studies will be aimed at identifying the individual components of the ERα/MAPK pathway as well as other pathways involved in the regulation of telomerase activity. Ultimately, a better understanding of the relationship between estrogen, MAPK signaling and telomerease activity may lead to the development of novel therapeutic and preventative strategies for endometrial cancer as well as other hormonally-driven cancers.
